# Ishemia-Reperfusion enhances GAPDH nitration in aging skeletal muscle

**DOI:** 10.18632/aging.100394

**Published:** 2011-10-23

**Authors:** C. Eric Bailey, David W. Hammers, James H. DeFord, Vincent L. Dimayuga, James K. Amaning, Roger Farrar, John Papaconstantinou

**Affiliations:** ^1^ Department of Biochemistry & Molecular Biology, University of Texas Medical Branch, Galveston, TX 77555-0643, USA; ^2^ Department of Kinesiology, The University of Texas at Austin, 1 University Station, Austin, TX 78712, USA

**Keywords:** Skeletal muscle, GAPDH, nitration, Ischemia/Reperfusion, aging

## Abstract

Aging and skeletal muscle ischemia/reperfusion (I/R) injury leads to decreased contractile force generation that increases severely with age. Our studies show that glyceraldehyde-3-phosphate dehydrogenase (GAPDH) protein expression is significantly decreased at 3 and 5 days reperfusion in the young mouse muscle and at 1, 3, 5, and 7 days in the aged muscle. Using PCR, we have shown that GAPDH mRNA levels in young and old muscle increase at 5 days reperfusion compared to control, suggesting that the protein deficit is not transcriptional. Furthermore, while total tyrosine nitration did not increase in the young muscle, GAPDH nitration increased significantly at 1 and 3 days reperfusion. In contrast, total tyrosine nitration in aged muscle increased significantly at 1, 3, and 5 days of reperfusion, with increases in GAPDH nitration at the same time points. We conclude that GAPDH protein levels decrease following I/R, that this is not transcriptionally mediated, that the aged muscle experiences greater oxidative stress, protein modification and GAPDH degradation, possibly contributing to decreased muscle function. We propose that tyrosine nitration enhances GAPDH degradation following I/R and that the persistent decrease of GAPDH in aged muscle is due to the prolonged increase in oxidative modification in this age group.

## INTRODUCTION

Skeletal muscle dysfunction associated with ischemia/reperfusion (I/R) injury is in part attributed to increased oxidative stress and oxidative protein modification [1-3] This oxidative stress is associated with mitochondrial ROS production, the inflammatory response resulting from the action of cytokines and the infiltration of inflammatory cells which increase in I/R during reoxygenation following reperfusion [1, 4].

Ischemia is a common problem in clinical practice. It is estimated that by 2030 there will be 1-2 million vascular surgery cases per year in the United States [5], and I/R is a major component of these cases [6]. In addition, utilization of flaps during reconstructive surgery, tourniquets during orthopedic surgery, compartment syndrome following surgery or trauma, sepsis, critical limb ischemia, and thromboembolic events can all lead to I/R injury [2, 7, 8]. Many of these clinical situations are more common in elderly patients, who may be experiencing age-related muscle dysfunction. As oxidative stress and protein modification play a role in aging and I/R injury, it might be expected that I/R injury would have a more severe effect on muscle function in the elderly. Recent research in a rodent model of I/R are consistent with this idea [2]; however, the degree to which oxidative modification may play a role in this cumulative effect and the mechanisms by which oxidative protein modification may lead to muscle dysfunction are not well understood.

The metabolic phenotype of muscle fibers directly influences their susceptibility to oxidative stress. During aging, there is a preferential decrease in fiber number and fiber size for glycolytic skeletal muscles [9, 10]. Oxidative stress appears to have a greater impact upon mitochondrial electron transport chain function in glycolytic skeletal muscles compared to oxidative muscles [11]. Furthermore, the degree of glycolytic phenotype in skeletal muscle correlates with susceptibility to cachexia as a result of chronic disease in humans and in models of cachexia both in mouse muscle *in vivo* or mouse cell lines [12]. It is suggested that this is the result of increased anti-oxidant defenses and inducible nitric oxide synthase. The increased susceptibility of primarily glycolytic muscle to oxidative stress is the rationale for studying glycolytic skeletal muscle in the present work, as this fiber type would likely be the most severely impacted by injury. Furthermore, we chose to study the effects of oxidative stress because of its central role in the regulation of the glycolytic pathway which is an important source of energy.

Nitric oxide has been reported to increase in I/R injury although the timing and duration of the increase vary with experimental conditions [3, 13-15]. While at specific concentrations nitric oxide may be a protective factor in certain tissue stress signaling processes, excess nitric oxide can be detrimental [12, 16]. For example, interactions of nitric oxide with ROS can lead to several different types of post-translational modifications (for reviews, see [17-19]. Thus, when combined with superoxide, nitric oxide leads to the formation of damaging products [20], whether peroxynitrite [21] or one of peroxynitrite's potential end products, tyrosine nitration [22]. While some nitrosative modifications such as cysteine nitrosylation have been shown convincingly to be involved in signaling mechanisms [23-25], tyrosine nitration is usually considered to be an indication of high oxidative and nitrative stress that results in protein damage. Nitrotyrosine adducts are formed *in vivo* by two primary reaction pathways. These include (a) the reaction of nitric oxide with superoxide leading to peroxynitrite production and (b) the reaction of nitrite and hydrogen peroxide with various heme peroxidases. Both of these reactions lead to formation of a tyrosyl radical, followed by addition of nitrogen dioxide to yield 3-nitrotyrosine [26]. In I/R injury, both mechanisms of tyrosine nitration are likely to be elevated, due to the infiltration of immune cells, activation of iNOS, and increased ROS production [3]. To examine the role of aging and oxidative stress in muscle dysfunction following I/R injury, we used a hind limb tourniquet model to induce I/R injury in young and old mice. GAPDH protein and mRNA levels were assessed to provide insight into the effects of I/R injury on glycolytic activity. Protein tyrosine nitration was evaluated, and in addition to GAPDH, several other nitrated proteins were identified via 2D gel electrophoresis and mass spectrometry.

## RESULTS

### The effect of I/R on GAPDH pool levels in *plantaris*/FHL from young and aged mice

**Figure 1:**
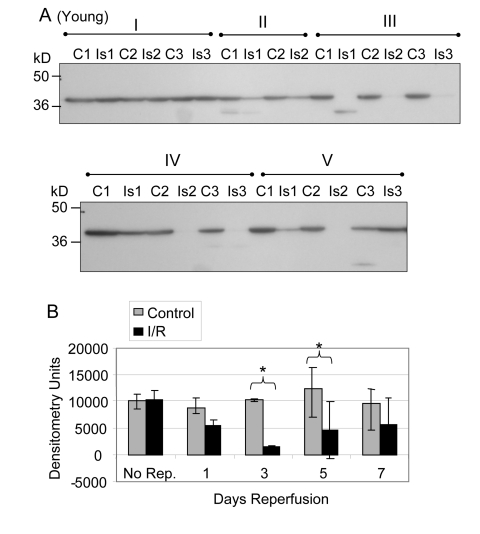
GAPDH pool levels in *plantaris/FHL* lysate from young mice Protein (30 μg) from total muscle cell lysates of plantaris/FHL from young mice (6-7 months) subjected to two hours of ischemia were separated by SDS-PAGE, transferred to PVDF membrane, and blotted with anti-GAPDH antibody. (**A**) Group I: No reperfusion, showed no significant difference. Group II: One day reperfusion shows that GAPDH levels decreased, but not significantly. Groups III and IV: Three and five days reperfusion, respectively, showed a significant reduction in GAPDH immunoreactive pool levels (p = 0.0004 and p = 0.007, respectively). Group V: Seven days reperfusion showed no significant difference between control and I/R muscle samples. (**B**) A bar graph showing the statistical differences in GAPDH pool levels in response to 2 hours of ischemia and 1-7 days of reperfusion.

**Figure 2:**
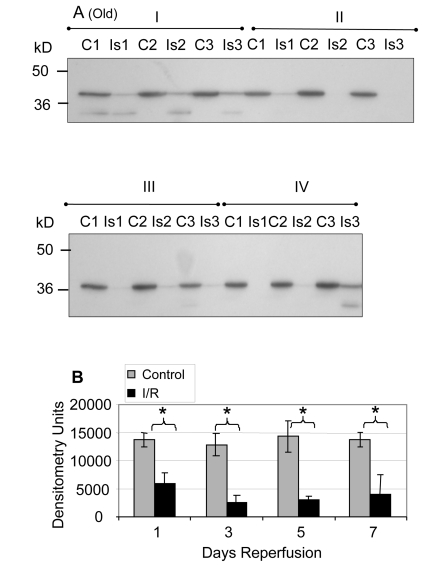
GAPDH pool levels in *plantaris/FHL* lysate from old mice Protein (30 μg) from total muscle cell lysates of *plantaris*/FHL from old mice (24-27 months) subjected to two hours of ischemia were separated by SDS-PAGE, transferred to PVDF membrane, and blotted with anti-GAPDH antibody. (**A**) Groups I, one day; II, 3 days, III, 5 days and IV, 7 days of reperfusion all showed statistically significant decreases in GAPDH immunoreactive pool levels. (**B**) A bar graph showing the statistical differences in GAPDH pool levels in response to 2 hours of ischemia and 1-7 days of reperfusion.

Glycolytic muscle mass decreases following I/R injury, particularly in muscle of old rats, suggesting a decrease in protein content [2]. Furthermore, the decrease in force production after I/R injury is greater for old muscle suggesting that decreased force production is related to decreased expression of glycolytic and contractile proteins [2]. Based on these observations we determined the protein pool levels of GAPDH for young and old mouse glycolytic skeletal muscle subjected to I/R injury (Figures 1, 2). Prior to normalization of the Western blot data, the comparison of GAPDH protein levels between young control and ischemic muscle showed no statistically significant difference after 2 hours ischemia with no reperfusion (Figure 1A, 1B Group I), after 1 day reperfusion (Group II), or after 7 days reperfusion (Figure 1A, 1B Group V). At the 3 and 5 day time points (Groups II and IV) the GAPDH protein levels were significantly decreased in the I/R tissue, with an average decrease of 89.4% (p = .0004) at 3 days and 71.4% (p = 0.007) at 5 days (Figure 1B).

Similar analyses of the GAPDH protein levels in old muscle showed a significant decrease after reperfusion at days 1 (Group I; 61.9%, p = 0.0005), 3 (Group II; 83.1%, p = 0.014), 5 (Group III; 83.3%, p = 0.007), and 7 (Group IV; 73.8%, p = 0.013) of reperfusion (Figure 2A, 2B). The no reperfusion time point was not available for the old animals.

Although statistically significant differences in GAPDH pool level were seen at several time points in both age groups, a confounding variable in the data was the question of protein degradation in the ischemic muscle as related to the I/R injury. An examination of the post-transfer gels after Coomassie staining revealed an increase in low molecular weight bands (< 16 kD) in the ischemic samples after reperfusion. Further, comparison of the total protein staining of the one-dimensional gel electrophoresis revealed that the average decrease of GAPDH over all days of reperfusion in the young was 16.2% (p = 0.003 compared to young control).

In the old muscle, there was a 16.6% decrease compared to old controls (p < 0.0001) as indicated by staining the I/R samples, after equalizing protein input prior to loading of the gels. Significantly, the samples from the young animals which had received 2 hours of ischemia but no reperfusion showed no statistically significant differences in protein loading between control and ischemic muscle. In order to correct for differences in the amount of protein actually present in the gel, a normalization factor based on the Coomassie staining of the post-transfer gels was used (as described in Methods).

After normalizing for background levels of protein degradation, the pool level of GAPDH in glycolytic skeletal muscle from young mice was still significantly decreased at 3 and 5 days of reperfusion (p = 0.001 and 0.005 respectively). There were no significant alterations at 2 hours of ischemia without reperfusion at day 1 or at day 7 of reperfusion. After normalization for protein degradation the GAPDH protein pool levels in skeletal muscle from the old mice were still significantly decreased at 1, 3, 5, and 7 days reperfusion (p = 0.001, 0.015, 0.011, and 0.007, respectively).

### Total RNA from control and ischemic *plantaris* following 5 days of reperfusion

**Figure 3:**
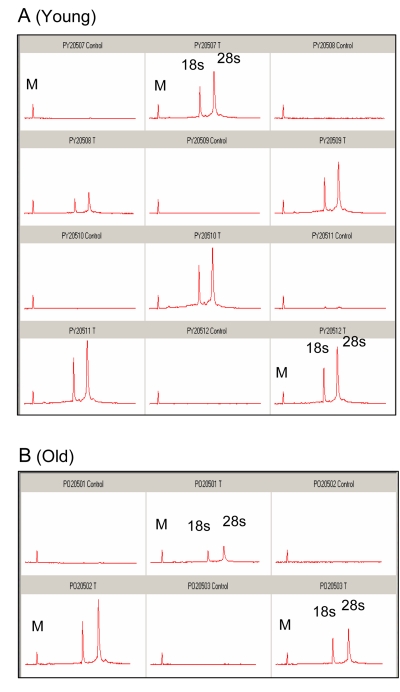
Analysis of total RNA from control and ischemic *plantaris* muscle following 5 days of reper-fusion An equal percentage of total RNA from (**A**) six separate samples of young (6-7 months) and (**B**) 3 separate samples of old (24-27 months) *plantaris* muscle was analyzed by capillary gel electrophoresis. The initial peak is a marker (M) included for calibration. Control and I/R samples are the contralateral and ischemic muscle from the same animal. Samples are paired from left to right, beginning with control. C = control; T = I/R; small ribosomal subunit (18s) and large ribosomal subunit (28s).

We examined the possibility that altered GAPDH transcription may play a role in the decrease in GAPDH pool levels. Total RNA from *plantaris* muscle was isolated from young and old mice subjected to 2 hours of ischemia and 5 days of reperfusion. An equal percentage of total RNA from each sample was analyzed using a chip-based gel electrophoresis assay to determine the quality of isolated RNA. Surprisingly, the yield of total RNA from the ischemic samples was substantially higher compared to the contralateral control, regardless of age (Figure 3A and B). The data indicate that the bulk of the increase in total RNA was from rRNA as measured by18s and 28s ribosomal subunits (Figure 3A and B).

### PCR analysis of GAPDH transcript levels from control and I/R *plantaris* following 5 days of reperfusion

**Figure 4:**
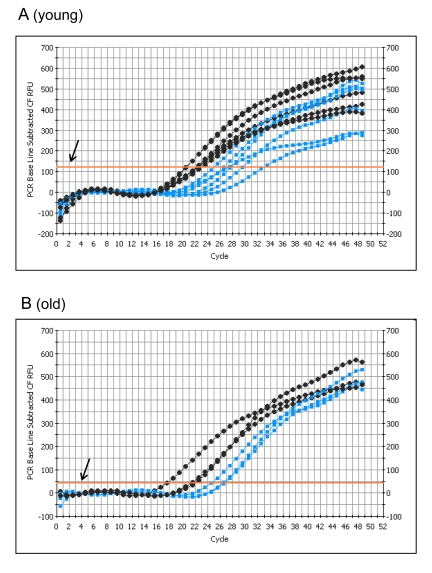
PCR analysis of GAPDH transcript levels from control and I/R *plantaris* muscle following 5 days of reperfusion PCR with GAPDH specific probes was performed using an equal percentage of total cDNA (reverse transcribed from mRNA using igo(d)T primers) isolated from each sample of (**A**) young (6-7 months) and (**B**) old (24-27 months) plantaris muscle. Control = black; I/R = gray; T = Threshold (arrow shows the threshold).

To determine whether mRNA levels are affected, GAPDH-specific primers were used to perform PCR on an equal percent of the total cDNA (Figure 4). Significant differences were detected in the number of cycles to threshold (Ct) between young ischemic and young controls, with an average difference of 6.2 cycles (p = 0.0009).

The difference between the old controls and old ischemics was also significant (p = 0.049), with an average difference of 5.4 cycles (Figure 4B). These data clearly show that there is a decrease in the GAPDH transcript levels in the I/R muscles. The difference in GAPDH transcript levels in response to I/R between the young and the old was not statistically significant. However, the data (threshold levels) suggest that the average level of GAPDH mRNA in the young muscle is approximately twice the level in the old (Figure 4).

### Detection of nitrotyrosine modified proteins

**Figure 5:**
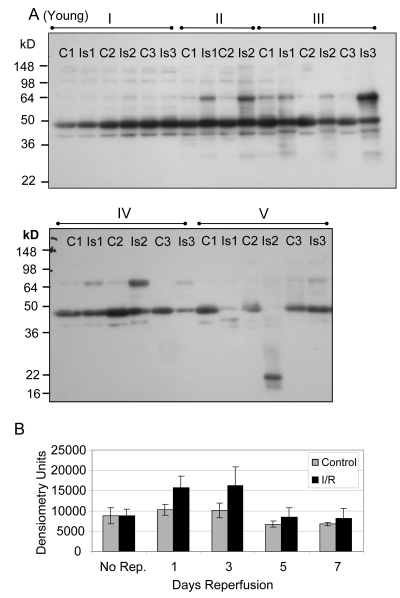
Western blot analysis of total tyrosine nitration of *plantaris/FHL* lysate from young mice following I/R (**A**) Tyrosine nitration of whole cell lysate of glycolytic skeletal muscle from young (6-7 months) mice with 2 hours ischemia and variable lengths of reperfusion was examined by Western blot. C = contralateral control; Is = I/R; Group I = no reperfusion; Group II = 1 day reperfusion; Group III = 3 days reperfusion; Group IV = 5 days reperfusion, and Group V = 7 days reperfusion. (**B**) Densitometric analysis shows no statistically significant differences in levels of overall nitration.

**Figure 6:**
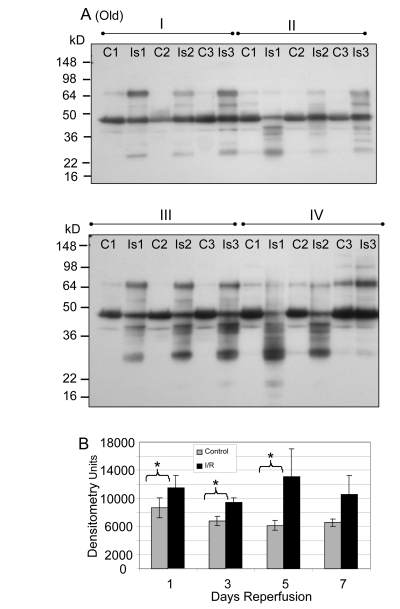
Western blot analysis of total tyrosine nitration of *plantaris/FHL* lysate from old mice following I/R (**A**) Tyrosine nitration of whole cell lysate of glycolytic skeletal muscle from old mice (24-27 months) with 2 hours ischemia and variable lengths of reperfusion was examined by Western blot. C = contralateral control; Is = I/R; Group I = 1 day reperfusion; Group II = 3 days reperfusion; Group III = 5 days reperfusion, and Group IV = 7 days reperfusion. (**B**) Densitometric analysis shows statistically significant differences were observed at 1, 3, and 5 days of reperfusion.

Previous studies have shown functional impairment of glycolytic skeletal muscle following I/R injury [2]. Oxidative modification of proteins critical for muscle function is a possible mechanism explaining the decline in muscle force production. As levels of reactants necessary to form nitrotyrosine are known to increase during the reperfusion stage of I/R injury, we tested the hypothesis that protein tyrosine nitration would increase following reperfusion. Relative levels of protein tyrosine nitration were assayed in skeletal muscle extract from young (Figure 5) and old (Figure 6) mice following I/R by one-dimensional gel electrophoresis and Western blotting with antibody specific for nitrotyrosine. The corresponding contralateral muscles were used as a control for the basal level of nitration (Figures 5, 6). Following normalization for back-ground protein degradation, there were no statistically significant differences in total nitrotyrosine for the young, although nitration was increased at every time point following reperfusion (Figure 5; range 22% to 67% increase).

In contrast to the young, an analysis of total tyrosine nitration in whole cell lysates of old skeletal muscle following I/R revealed statistically significant increases at the 1, 3, and 5 day time points (Figure 6; p = 0.008, 0.048 and 0.03, respectively). Total nitration was increased at all time points, but the increase was not significant at the 7 day time point (Figure 6).

While evaluating overall levels of an oxidative modification is useful as an indication of oxidative stress in a tissue, knowing which specific proteins are targeted by posttranslational modifications can further provide an understanding of the consequences of the modifications. Therefore, experiments were undertaken to identify those proteins which were targets of tyrosine nitration in I/R injury. Two-dimensional gel electrophoresis was used to resolve lysate proteins from young (6 months) and old (24-27 months) muscles previously assayed by one dimensional gel electrophoresis and western blot (Figure 7). The five day reperfusion time points were selected for analysis. Anti-nitrotyrosine western blotting was used to select spots from a second gel of the same sample (focused concurrently with the gel used for Western blotting) for identification via MALDI-TOF mass spectrometry. A list of identified proteins is included in Table 1. Protein scores greater than 53 were considered to be significant. Of the proteins identified by mass spectrometry in the old muscle following I/R, approximately 30% were also identified in the young following I/R. It was noted that several enzymes in the glycolytic pathway were identified as nitrated, based on Western blot selection for MALDI-TOF. Three of these enzymes, GAPDH, triose phosphate isomerase, and fructose bis-phosphate aldolase A, were identified from both young and old. Since glycolysis is necessary to maintain adequate ATP levels during I/R injury, any effect on the activities of these enzymes might have a significant effect on ATP levels and consequently on energy availability.

**Table 1:** Identification of 3-Nitrotyrosine Modified Proteins by Mass Spectrometry

#	Protein Name (Mus Musculus)	Protein MW	Peptide Count	Protein Score	Protein PI	Expectation Score
**Young - 5 Days Reperfusion**						
1	Aconitate hydratase, mitochondrial precursor	85,410.0	13	70	8.08	1.26E-03
2	Actin, alpha cardiac muscle 1	41,991.9	10	450	5.23	1.26E-41
3	Actin, alpha skeletal muscle	42,023.9	12	482	5.23	7.92E-45
4	Actin, cytoplasmic 2	41,765.8	7	220	5.31	1.26E-18
5	Actin, gamma-enteric smooth muscle	41,849.8	8	304	5.31	5.00E-27
6	Annexin A11	54,076.8	11	146	7.53	3.15E-11
7	ATP synthase subunit alpha, mitochondrial precursor	59,715.6	17	290	9.22	1.26E-25
8	Carbonic anhydrase	29,347.6	5	57	6.89	2.51E-02
9	Carbonic anhydrase 3	29,347.6	8	183	6.89	6.29E-15
10	Creatine kinase M-type	43,017.8	16	562	6.58	7.92E-53
11	Desmin	53,456.0	12	67	5.21	2.51E-03
12	Dihydrolipoyl dehydrogenase, mitochondrial precursor	54,238.2	10	269	7.99	1.58E-23
13	ETS-related transcription factor Elf-3	44,245.6	10	59	5.75	1.58E-02
14	Fructose-bisphosphate aldolase A	39,331.3	10	108	8.31	1.99E-07
15	Glutamate dehydrogenase 1, mitocondrial precursor	61,298.2	8	75	8.05	3.97E-04
16	**Glyceraldehyde-3-phosphate dehydrogenase**	**35,787.2**	**9**	**180**	**8.44**	**1.26E-14**
17	Glycerol-3-phosphate dehydrogenase [NAD+], cytoplasmic	37,548.4	13	311	6.75	9.98E-28
18	Heat shock protein beta-1	22,999.7	8	254	6.12	5.00E-22
19	Malate dehydrogenase, cytoplasmic	36,488.1	7	188	6.16	1.99E-15
20	NADH dehydrogenase [ubiquinone] flavoprotein 2, mitochondrial precursor	27,267.9	4	54	7	5.00E-02
21	Nebulin-related-anchoring protein	195,651.6	23	59	9.35	1.58E-02
22	Peroxiredoxin-6	24,855.0	5	149	5.71	1.58E-11
23	Pyruvate kinase isozymes M1/M2	57,808.0	13	128	7.18	1.99E-09
24	Rho GDP-dissociation inhibitor 1	23,392.8	11	270	5.12	1.26E-23
25	Serum albumin precursor	68,647.7	5	207	5.75	2.51E-17
26	Triosephosphate isomerase	26,695.8	6	199	6.9	1.58E-16
27	UTP-glucose-1-phosphate uridylyltransferase	56,943.7	11	67	7.18	2.51E-03
**Old - 5 Days Reperfusion**						
#	Protein Name (Mus Musculus)	Protein MW	Peptide Count	Protein Score	Protein PI	Expectation Score
1	Actin, alpha cardiac muscle 1	41,991.9	4	98	5.23	1.99E-06
2	Actin, alpha skeletal muscle	42,023.9	4	97	5.23	2.51E-06
3	Actin, cytoplasmic 1	41,709.7	8	134	5.29	5.00E-10
4	Actin, cytoplasmic 2	41,765.8	2	57	5.31	2.51E-02
5	Actin, gamma-enteric smooth muscle	41,849.8	3	63	5.31	6.29E-04
6	Ankyrin repeat domain-containing protein 49	27,094.5	7	54	5.04	5.00E-02
7	Annexin A11	54,076.8	10	299	7.53	1.58E-26
8	Apolipoprotein A-1 precursor	30,568.7	17	622	5.64	7.92E-59
9	ATP synthase subunit alpha, mitochondrial precursor	59,715.6	16	383	9.22	6.29E-35
10	Creatine kinase M-type	43,017.8	13	456	6.58	3.15E-42
11	Fibrinogen beta chain precursor	54,717.7	8	90	6.68	1.26E-05
12	Fructose-bisphosphate aldolase A	39,331.3	5	68	8.31	1.99E-03
**13**	**Glyceraldehyde-3-phosphate dehydrogenase**	**35,787.2**	**5**	**150**	**8.44**	**1.26E-11**
14	Ig gamma-2B chain C region membrane-bound form	44,231.2	3	174	6.1	3.00E-14
15	Ig gamma-2B chain C region secreted form	36,635.3	3	175	7.19	3.97E-14
16	Ig kappa chain C region	11,770.5	2	68	5.23	1.99E-03
17	Lamin-A/C	74,192.7	7	72	6.54	7.92E-04
18	L-lactate dehydrogenase A chain	36,475.2	7	117	7.62	2.51E-08
19	Myosin-1	223,203.0	8	161	5.6	9.98E-13
20	Myosin-3	223,652.4	8	121	5.62	9.98E-09
21	Myosin-4	222,719.9	9	264	5.58	5.00E-23
22	Myosin-6	223,426.5	24	441	5.57	9.98E-41
23	Myosin-7	222,740.5	28	537	5.59	2.51E-50
24	Myosin-8	222,569.5	10	231	5.65	9.98E-20
25	Nucleoside diphosphate kinase B	17,351.9	4	60	6.97	1.26E-02
26	Peptidyl-prolyl cis-trans isomerase A	17,959.8	2	55	7.74	3.97E-02
27	Phosphoglycerate mutase 1	28,813.9	8	175	6.67	3.97E-14
28	Phosphoglycerate mutase 2	28,808.8	4	103	8.65	6.29E-07
29	Putative RNA-binding protein 3	16,594.7	8	258	6.84	1.99E-22
30	Serotransferrin precursor	76,673.7	13	294	6.94	5.00E-26
31	Serum albumin precursor	68,647.7	5	124	5.75	5.00E-09
32	Strumpellin	134,024.8	12	57	6.72	2.51E-02
28	Superoxide dismutase [Cu-Zn]	15,932.8	9	406	6.02	3.15E-37
29	Transthyretin precursor	15,766.0	9	446	5.77	3.15E-41
30	Triosephosphate isomerase	26,695.8	6	246	6.9	3.15E-21
31	Tropomyosin alpha-1 chain	32,660.7	26	687	4.69	2.51E-65
32	Tropomyosin alpha-3 chain	32,842.8	13	206	4.68	3.15E-17
33	Tropomyosin alpha-4 chain	28,450.4	6	68	4.65	1.99E-03
34	Tropomyosin beta chain	32,816.6	18	275	4.66	3.97E-24

**Figure 7:**
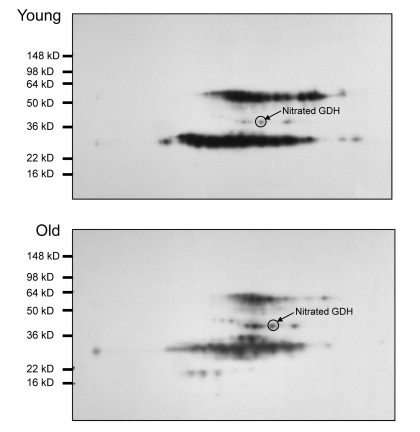
Nitrotyrosine western blot analysis of 2D gel electrophoresis of young and old *plantaris/FHL* lysate following 5 days of reperfusion *Plantaris/FHL* lysates (180 μg) from (**A**) young (6-7 months) or (**B**) old (24-27 months) I/R tissues were separated by 2D gel electrophoresis. Nitrotyrosine immunoblotting was used to select nitrotyrosine modified proteins from a duplicate gel for mass spectrometry and identification.

### Identification of nitrated GAPDH in young and old muscle

After identifying the nitrated proteins from I/R samples, we compared the 37 kD band corresponding to the molecular weight of GAPDH across the time course. We focused on GAPDH due to its importance in glycolysis, reports of altered GAPDH function with posttranslational modification *i.e.*, nitrosylation [23], and a recent report that overexpression of GAPDH can rescue cells in culture from caspase-independent cell death [27]. These reports suggest that GAPDH has multiple functions and may integrate different cellular activities with the metabolic state of the cell [28].

**Figure 8:**
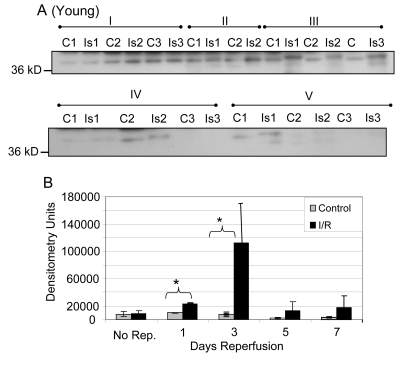
Tyrosine nitration of GAPDH in *plantaris/FHL* lysate from young mice (**A**) Tyrosine nitration was assessed by Western blot of one dimensional gel electrophoresis. (**B**) Densitometric analysis shows nitration was significantly increased for GAPDH at 1 and 3 days of reperfusion, with the greatest increase at 3 days reperfusion. C = contralateral control; Is = I/R; Group I = no reperfusion; II = 1 day reperfusion; Group III = 3 days reperfusion; Group IV = 5 days reperfusion, and Group V = 7 days reperfusion. * = p < 0.05.

After normalizing the nitration of the 37 kD band to GAPDH protein pool levels as measured by Western blotting, it was found that the nitration of GAPDH in the young was significantly increased at the one and three day time points (Figure 8; p = 0.001 and 0.043 respectively). The highest level of nitration in the samples from young mice occurs at the 3 day time point.

**Figure 9:**
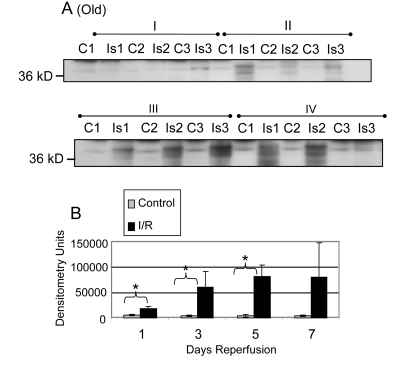
Tyrosine nitration of GAPDH in *plantaris/FHL* lysate from old mice (**A**) Tyrosine nitration was assessed by Western blot of one dimensional gel electrophoresis. (**B**) Densitometric analysis shows nitration was significantly increased for GAPDH at 1, 3, and 5 days of reperfusion. C = contralateral control Is = I/R; Group I = 1 day reperfusion; Group II = 3 days reperfusion; Group III = 5 days reperfusion, and Group IV = 7 days reperfusion. * = p < 0.05.

After normalization to GAPDH pool level, the nitration of the 37 kD band in the samples from old mice was significantly increased at 1, 3, and 5 days reperfusion (Figure 9; p = 0.019, 0.048, and 0.011, respectively). In contrast to the young, the highest levels of nitration measured were at the 5 and 7 day time points. However, the increase in nitration was not statistically significant at 7 days due to the variability within the I/R group (Figure 9).

Comparing the percent increase in nitration (control to I/R) of the 37 kD band, a significant difference (p = 0.02) was noted between the young and old skeletal muscle at the 5 day time point corresponding to the peak of GAPDH nitration in the old. The level of nitration in the young had decreased by the 5^th^ day with the peak of the increase in nitration appearing at the 3^rd^ day of reperfusion. In summary, the greatest difference between control and I/R in the young occurs at 3 days of reperfusion, while the greatest difference in the old occurs at 5 days of reperfusion.

## DISCUSSION

We have presented evidence that following I/R, GAPDH protein levels decrease in both young and old mouse skeletal muscle while levels of nitrated GAPDH increase. Our observations are consistent with the reports that oxidative protein modifications increase protein degradation [29, 30] and suggest a model of protein homeostasis in which increased oxidative modification and subsequent degradation of proteins leads to decreases in protein pool levels.

Our experiments show evidence of increased proteolytic products in the I/R samples in both young and aged, in the form of low molecular weight products in the post-transfer gels. Thus, increased tyrosine nitration of GAPDH following I/R injury in both the young and the old muscle may lead to protein unfolding and increased susceptibility to aggregation or degradation [31, 32] which is one of several factors that may affect the protein pool level following I/R. We thus propose that tyrosine nitration, as an oxidative modification, may signal increased degradation by the 20S proteasome compared to the unmodified enzyme [30] or the chaperone-mediated autophagy pathways [29, 30, 33]. Specifically, *in vitro* tyrosine nitration increases the rate of enzyme degradation which is a potential mechanism for the decrease in GAPDH pool levels. The fact that GAPDH nitration remains elevated for a longer period of time in the old compared to young muscle, *i.e.*, up to 7 days supports our hypothesis that (a) oxidative stress and oxidative protein modification in aging and I/R are cumulative, and that (b) I/R injury, therefore, has a proportionately greater impact upon the old muscle, where antioxidant defenses are already taxed by chronically elevated ROS. As increased levels of nitration correlate with decreases in GAPDH pool levels for both age groups, it further suggests that oxidative modification may be driving proteolytic degradation of GAPDH following I/R injury.

The decrease in GAPDH mRNA levels during reperfusion does not account for the deficit in GAPDH protein levels. Furthermore, the increase in rRNA may be a compensatory response to the decreased protein levels. The question that remains, however, is why are GAPDH protein levels so severely decreased in the presence of both mRNA and rRNA transcripts? Our results thus suggest an alternative mechanism that involves dysfunctional or decreased GAPDH translational processes. This possibility is supported by reports that IGF-1 mRNA is upregulated following I/R, suggesting that protein translation should be increased [2, 5].

Histological analyses (Hammers et al., personal communication) have shown significant levels of inflammatory cell infiltration at 5 days in both age groups. These inflammatory cells may contribute to the increase in total RNA seen in the I/R samples. Further studies should fully elucidate the contribution made by immune cells in the current results.

The increase in rRNA following ischemia and 5 days reperfusion may provide increased capacity for protein synthesis. This is in contrast, however, to what has been reported in rats, where no difference was seen in the level of 18S rRNA in gastrocnemius at 7 days [2]. However, it should be noted that in addition to the differences in muscle choice, species, and time point, different experimental procedures may explain the discrepancy between these results. In the study by Hammers and Farrar [2], total RNA content was normalized prior to reverse transcription in accordance with accepted procedure. In our experiments, total RNA quality was assayed prior to balancing. After detecting consistently disparate amounts of total RNA between I/R and control we reasoned that the differences were not due to the slight variation in starting muscle weight or to random variation in the isolation procedure. We decided, therefore, that balancing the total RNA would eliminate true differences in rRNA abundance and obscure variation in mRNA abundance. As rRNA constitutes the greatest part of total RNA, one could potentially create a paradoxical and artifactual decrease in a specific mRNA if the level of that mRNA was increased to a lesser extent than the rRNA. We therefore continued to examine equal percentages of the total RNA isolated.

Our findings that the rRNA is increased agree with the report that local insulin-like growth factor-1 (IGF-1) mRNA is increased in rat gastrocnemius following 2 hours of ischemia and 7 days of reperfusion [2]. IGF-1 has been reported to increase the transcriptional activity of RNA polymerase I in HEK293 cells to allow increased capacity for protein synthesis [34]. Increases in local IGF-1 have been reported following muscle injury in several different models [35, 36] including I/R hypoxia/reoxygenation [35]. The mechanism for this stimulation of ribosomal transcription is primarily via activation of the PI3K/Akt/mTOR pathway, although the Ras-MAPK pathway also contributes in HEK293 cells [34].

Another possible mechanism for the decrease in GAPDH pool level following I/R is a deficient or dysfunctional translation. Thus, the severe decrease in the GAPDH pool levels of the young in the presence of GAPDH transcript suggests that dysfunction of translation may also be involved in the young following I/R [36, 37]. Our results argue that an examination of translation would be an important step in understanding the observed phenomena. It would also be important to examine the changes in GAPDH transcript at earlier time points, particularly in the young to determine when the level of GAPDH transcript is altered, whether this contributes to decrease in the protein level or whether translation may be impaired. There is a precedent for translational arrest following I/R injury from studies examining cerebral [38, 39] and kidney I/R injury [40], although to our knowledge this has not been extended to skeletal muscle. In summary, the effects of I/R on GAPDH mRNA and rRNA do not adequately explain the decrease in GAPDH protein levels, suggesting alterations in protein homeostasis during recovery from I/R injury.

Our studies raise the question of whether tyrosine nitration of GAPDH in I/R injury affects its enzymatic activity. It is unlikely that tyrosine nitration results in the initial loss of GAPDH activity based on the report that the loss of glycogen phosphorylase due to cysteine modification occurs prior to the detection of nitration [30, 41]. This is consistent with studies showing that: (a) mutation of cysteines in the GAPDH active site abolishes GAPDH glycolytic activity [27]; (b) oxidative modification by numerous agents results in loss of activity following modification of these active site cysteines, suggesting that cysteine oxidation following I/R injury in young and aged muscle may be an important physiological factor [42-45].

Our studies raise the question as to what extent our findings might be generalized to muscle fibers with a more oxidative phenotype. Metabolic phenotype strongly correlates with sensitivity to oxidative stress [11, 12, 46, 47]. As skeletal muscle with an oxidative phenotype is believed to be more resistant to oxidative stress, a comparison of GAPDH levels within predominately oxidative and predominately glycolytic muscles following I/R would provide a means of testing the hypothesis that oxidative stress and protein modification plays a major role in the observed decrease in GAPDH pool levels.

Another potential consequence of decreased GAPDH expression is that loss of GAPDH could sensitize the surviving muscle to further insults such as ROS/RNS from a prolonged inflammatory response seen in old rats following I/R injury [2]. Furthermore, the loss or gain of sensitivity is a function of the level of GAPDH expression. This is consistent with the observation that over-expression of GAPDH can prevent caspase-independent cell death in response to treatment with etoposide, staurosporine, or actinomycin D [27]; that chronic myelogenous leukemia (CML) cells resistance to imatinib therapy spontaneously upregulated GAPDH expression and that these resistant cells could be sensitized by siRNA knockdown of GAPDH expression [48].

The central observation of this study is that GAPDH protein levels are decreased in both old and young muscle following I/R injury. Our data suggest a model of protein homeostasis following I/R injury in which increased oxidative modification and subsequent degradation of proteins leads to decreases in protein pool levels. Cessation of oxidative stress would, therefore, allow transcription and translation to return protein pools to normal. This is consistent with our observation that the declining levels of oxidative modification in the young correlate with the normalization of GAPDH pool levels whereas for the old animals normalization of GAPDH pool levels is significantly delayed, *i.e.*, were not reached by 7 days in spite of evidence for increased GAPDH transcription. Thus, while the young have begun to recover the GAPDH pool level, either the prolonged oxidative stress and oxidative modification in the old mouse continues to drive protein degradation or that translation is impaired. Although our data support the hypothesis that degradation is responsible for the decreased GAPDH pool levels, we do not discount a significant role for decreased translation.

## METHODS

### Animals and tissues

Young (6-7 months) and old (24-27 months) C57Bl/6 mice were used in these experiments. Prior to their use, the mice were acclimatized to their new surroundings for one week. Upon completion of the experiments, animals were euthanized using sodium pentobarbital. For one-dimensional gel electrophoresis and Western blots, 3 animals were used per time point, with the exception of the young at 1 day, which had 2 animals. *Plantaris* and *flexor hallicus longus (FHL)* muscles were collected for the one dimensional gels. Time points examined for young and old mice were 2 hours of ischemia followed by either 1, 3, 5, or 7 days of reperfusion. For the young mice, an additional time point with 2 hours of ischemia and no reperfusion was also examined. For two dimensional gel electrophoresis, a representative sample from the 5 day time point was selected from the young and the old samples. For RNA extraction and PCR analysis, *plantaris* muscles from 6 young and 3 old animals were collected after 2 hours of ischemia followed by 5 days of reperfusion. All experimental procedures were approved by and conducted in accordance with guidelines set by the University of Texas at Austin IACUC.

### Ischemia/Reperfusion protocol

Tourniquet-induced I/R was performed as previously described [2]. Mice were anesthetized using 2% isofluorane prior to and for the duration of the tourniquet application. A heat lamp was used to maintain the animal's body temperature at 32-35 ± 1°C during the tourniquet procedure. After 2 hours, the tourniquet was removed, and the animal returned to its cage to recover. After the appropriate length of reperfusion, the muscles were taken, snap frozen in liquid nitrogen, and the samples were stored at −80°C until analysis.

### Protein extraction

*Flexor halicus longus* and *plantaris* muscles were homogenized together in 2D lysis buffer (8 M urea, 4% CHAPS, 40 mM DTT, protease inhibitors) using the Ettan Sample grinding kit (Amersham BioSciences), according to manufacturer's instructions. Briefly, the samples were homogenized in the sample grinding tube with abrasive resin by using the provided plastic pestles. The samples were centrifuged for 5 minutes at 10,000g to pellet the resin and debris. Supernatant was removed to a fresh tube. Additional 2D lysis buffer was added and the previous steps repeated to maximize yield. In some experiments supernatants were combined.

### Protein assays

Bradford protein assays (BioRad) of muscle homogenates were performed in triplicate using bovine serum albumin as a protein standard. The Bradford assays were performed in triplicate after the samples were homogenized, and the average value used. The samples to an equal concentration. The samples were then diluted, aliquoted and frozen at −80°C. until further use.

### One dimensional gel electrophoresis

Muscle samples (30 μg) were mixed with 5X Laemli loading buffer (10% SDS, 500 mM DTT, 300 mM Tris, 50% glycerol, 0.05% bromophenol blue, pH 6.8) and brought up to 25 μL with 2D lysis buffer. Samples were separated on a 4-20% tris-glycine gradient gel (Bio-Rad) at 135 volts for 2 hours in SDS running buffer (25 mM Tris base, 0.1% SDS (w/v), 192 mM glycine). Immobilon PVDF membranes (Millipore) were prepared by wetting the membrane for 15 seconds in methanol, equilibrating the membrane in distilled water for 2 minutes, and then equilibrating the membrane in transfer buffer (25 mM Tris base, 10% methanol (v/v), 192 mM glycine) for 5 minutes. Gel transfers were performed in a Hoeffer wire transfer tank at 50 volts for 2 hours at 4°C.

### Two dimensional gel electrophoresis

Muscle samples (180 μg) isolated in 2D lysis buffer were mixed with Destreak reagent (GE -BioSciences) plus 0.75% IPG 3-10 pH IPG buffer. Samples were made up to 200 μl with 2D lysis buffer. The sample was pipetted into an 11 cm Ettan IPGphor strip holder (Amersham Biosciences) and overlaid with an 11 cm Immobiline Drystrip 4-7 pH gel strip (GE Biosciences) with the backing strip of the gel toward the top. Immobiline DryStrip cover fluid (Amersham BioSciences) was pipetted over the strip and sample to prevent evaporation, the cover placed on the holder, and the assembly rehydrated on an Ettan IPGphor isoelectric focusing system (Amersham Biosciences). Conditions used were as follows: 12 hours in-gel rehydration at 20°C with a constant current of 50 mA/strip followed by 3 focusing steps – 500V for 50 Vh, 1000V for 1000Vh and 8000V for 16000Vh.

Strips were equilibrated for 15 minutes in SDS equilibration buffer (6 M urea, 29.3% glycerol (v/v), 2% SDS (w/v), 0.002% bromophenol blue (w/v), and 75 mM Tris-HCl, pH 8.8. Prior to equilibration, DTT (1% w/v) was added to the SDS equilibration buffer. The gel strip was then placed in the sample lane of a 10-20% Tris-HCl IPG +1 well, 1 mm Criterion gel (BioRad) and sealed with agarose sealing solution (25 mM tris, 192 mM glycine, 0.1% SDS (w/v), 0.5% agarose, and 0.002% (w/v) bromophenol blue. After the agarose solidified the gels were placed in a Criterion running tank (BioRad), filled with running buffer (25 mM Tris base, 0.1% SDS (w/v), and 192 mM glycine). The sample was separated at 135 volts for approximately 2 hours. The gel was transferred using a Criterion blotter system (BioRad) with conditions as described for transfer of 1D electrophoresis gels.

### Western blotting

Membranes were blocked for one hour with 5% non-fat dried milk TBST (50.0 mM tris, 200 mM NaCl, 0.05% Tween-20, pH 7.4) and probed overnight with anti-nitrotyrosine antibody 1:2000 5% milk, TBS-T. Membranes were washed 3X for 5 minutes in TBS-T and probed with anti-mouse secondary antibody at RT for 1 hour. Prior to blotting membranes were washed 2X 5 minutes and 1X 15 minutes in TBS-T. The membrane was then incubated with Immobilon blot substrate (Millipore) for 5 minutes and exposed to film (Biomax MR, Kodak). Bands were assessed by densitometry (FluorChem 8900, AlphaInnotech). Membranes were stripped (Restore Western Blot Stripping Buffer, Pierce), reprobed with anti-mouse antibody to insure original primary anti-nitrotyrosine removal and then probed with GAPDH antibody (clone CA6, Santa Cruz) 1:5000 in 5% milk, TBST for 1 hour.

### Protein normalization

Total protein per lane was assessed by densitometry (FluorChem 8900, AlphaInnotech) with automatic background subtraction and a value of 100 was assigned to the lane with the highest density. The remaining lanes were expressed as a decimal fraction of the 100 lane. The reciprocal of the decimal was multiplied by the values generated from the Western data. For nitration of the 37 kD band, a ratio computed from the GAPDH western blot was used to normalize the amount of GAPDH present rather than total protein. A correction factor was also used between gels of the same age group by rerunning samples in separate gels and taking a ratio of the 37 kD bands in the first and second gels. The average of the ratios was used as the correction factor. For nitrotyrosine blots the ratio was based on total nitration of repeated samples.

### Sample preparation for mass spectrometry

Preparation of samples for mass spectrometry were performed by the Mass Spectrometry Core Facility at University of Texas Medical Branch, Galveston. Gel samples were cut into 1 mm size pieces or smaller and placed into separate 0.5 mL polypropylene tubes. Ammonium bicarbonate buffer (50 mM; 100 μl) was added to each tube and the samples were then incubated at 37°C for 30 minutes. After incubation the buffer was removed and 100 μl of water was added to each tube. The samples were then incubated again at 37°C for 30 minutes. After incubation the water was removed and 100 μl of acetonitrile was added to each tube to dehydrate the gel pieces. The samples were vortexed and after 5 minutes the acetonitrile was removed. Acetonitrile (100 μl) was again added to each of the sample tubes, vortexed and removed after 5 minutes. The samples were then placed in a speedvac for 45 minutes to remove excess solvent. Lyophilized trypsin (20 μg; Promega Corp.) was dissolved by adding 2 mL of 25 mM ammonium bicarbonate (pH 8.0). The trypsin solution was then vortexed and added to each sample tube in a minimal amount (~10 μL), *i.e.*, just enough to cover the dried gel. The samples were then incubated at 37°C for 6 hrs. After digestion the samples were removed from the 37° C oven and 1 μL of sample solution was spotted directly onto a MALDI target plate and allowed to dry. Alpha-cyano-4-hydroxycinnamic acid (1 μL; Aldrich Chemical Co.) matrix solution (50:50 acetonitrile/water at 5 mg/mL) was then applied on the sample spot and allowed to dry. The dried MALDI spot was further dried with compressed air (Decon Laboratories, Inc.) before inserting into the mass spectrometer.

### Mass spectrometry

Matrix-Assisted Laser Desorption Ionization Time-of-Flight Mass Spectrometry (MALDI TOF-MS) was used to analyze the samples and determine protein identity. Data were acquired with an Applied Biosystems 4800 MALDI TOF/TOF Proteomics Analyzer. Applied Biosystems software package included 4000 Series Explorer (v. 3.6 RC1) with Oracle Database Schema Version (v. 3.19.0) and Data Version (3.80.0). The software was used to acquire both MS and MS/MS spectral data. The instrument was operated in positive ion reflectron mode with a mass range of 850 – 3000 Da, and the focus mass was set at 1700 Da. For MS data, 1000-2000 laser shots were acquired and averaged from each sample spot. Automatic external calibration was performed using a peptide mixture with reference masses 904.468, 1296.685, 1570.677, and 2465.199.

Following MALDI-MS analysis, MALDI-MS/MS was performed on several (5-10) abundant ions from each sample spot. A 1kV positive ion MS/MS method was used to acquire data under post-source decay (PSD) conditions. The instrument precursor selection window was ±3 Da. For MS/MS data, 2000 laser shots were acquired and averaged from each sample spot. Automatic external calibration was performed using reference fragment masses 175.120, 480.257, 684.347, 1056.475, and 1441.635 (from precursor mass 1570.700).

Applied Biosystems GPS Explorer^TM^ (v. 3.6) software was used in conjunction with MASCOT to search the respective protein database using both MS and MS/MS spectral data for protein identification. Protein match probabilities were determined using expectation values and/or MASCOT protein scores. MS peak filtering included the following parameters: mass range 800 Da to 4000 Da, minimum S/N filter = 10, mass exclusion list tolerance = 0.5 Da, and mass exclusion list (for some trypsin and keratin-containing compounds) included masses 842.51, 870.45, 1045.56, 1179.60, 1277.71, 1475.79, and 2211.1. For MS/MS peak filtering, the minimum S/N filter = 10.

For protein identification, the murine taxonomy was searched in the SwissProtein database. Other parameters included the following: selecting the enzyme as trypsin; maximum missed cleavages = 1; fixed modifications included carbamidomethyl (C) for 2-D gel analyses only; variable modifications included oxidation (M); precursor tolerance was set at 0.2 Da; MS/MS fragment tolerance was set at 0.3 Da; mass = monoisotopic; and peptide charges were only considered as +1. The significance of a protein match based on both the peptide mass fingerprint (PMF) in the first MS and the MS/MS data from several precursor ions, is based on expectation values; each protein match is accompanied by an expectation value. The expectation value is the number of matches with equal or better scores that are expected to occur by chance alone. The default significance threshold is p < 0.05, so an expectation value of 0.05 is considered to be on this threshold. We used a more stringent threshold of 10^−3^ for protein identification (a lower expectation value represents a more significant score).

### RNA extraction

Total RNA extraction was accomplished using the RNAqueous-4PCR kit (Applied Biosystems, U.S.) according to manufacturer's instructions. *Plantaris* muscle was collected from young (n = 6) and old (n = 3) mice subjected to 2 hours of ischemia and 5 days of reperfusion. The muscle samples were snap-frozen in liquid nitrogen and stored at −80° C until use. The *plantaris* was cut in half on dry ice. One half of the muscle was transferred to a microfuge tube and homogenized by hand in the lysis/binding solution supplied with the kit using a plastic pestle. An equal volume of 64% ethanol was added to the solution and mixed. This mixture was pipetted into a tube containing a filter cartridge supplied with the kit. The cartridge and solution were centrifuged at 15,000g for 1 minute. Wash solution #1 was added to the retentate, and the tube was centrifuged as in the previous step. The previous wash and centrifugation was repeated twice using wash solution #2/3. The filter cartridge was placed into a new tube, and elution buffer pre-warmed to 70-80°C was added. The cartridge and tube were centrifuged at 15,000g for 1 minute. Another volume of heated elution buffer was added, and the cartridge was centrifuged again. Finally, the cartridge was removed and the total filtrate was centrifuged again. The supernatant was removed to a fresh tube, leaving any glass fibers from the cartridge in the pellet. Residual amounts of DNA were removed by treating the samples with DNase 1 provided in the RNAqueous kit. The samples were incubated at 37°C for 20 minutes, and DNase inactivation reagent was added. The samples were then gently vortexed, incubated with the inactivation reagent for 2 minutes and then centrifuged at 10,000g for 1 minute. The supernatant was then transferred to fresh tubes.

To ascertain the quality of the extracted mRNA, the rRNA 6000 Nano Assay kit (Agilent Technologies, Germany) was used according to manufacturer's instructions, in conjunction with the Agilent 2100 bioanalyzer. This chip-based capillary gel electrophoresis unit utilizes a proprietary dye-based reagent that binds to nucleic acids for detection and quantitation of rRNA.

### cDNA synthesis

Synthesis of cDNA was done using the SuperScript First-Strand Synthesis System for RT-PCR (Invitrogen) according to manufacturer's instructions. For each sample, an equal volume (8 μl) of DNAse-treated total RNA, 1 μl of 10 mM dNTP mix, and 1 μl of 0.5 μg/μl oligo(dT)_12-18_ was added to one well of a PCR reaction plate. A 2X reaction mixture was prepared which contained the following: 2 μl of 10X reaction buffer, 4 μl of 25 mM MgCl_2_, 2 μl of 0.1M DTT, and 1 μl RNaseOUT (provided in the kit). The RNA/primer mix was incubated at 65°C for 5 minutes using an Applied Biosystems GeneAmp 9700. The mixture was then placed on ice for 1 minute. Nine μl of the 2X reaction mixture was added to each reaction and mixed by pipetting. The reaction was incubated at 42°C for 2 minutes in the Applied Biosystems GeneAmp 9700 prior to the addition of 1μl of SuperScript II reverse transcriptase to each reaction (with the exception that no reverse transcriptase was added to the negative control). The reaction plate was incubated at 42°C for 50 minutes in the GeneAmp 9700. Subsequently, the temperature was increased to 70°C for 15 minutes to terminate the reaction. The reaction plate was chilled on ice for 5 minutes. RNase H (1μl) was added to each well and the plate was incubated at 37 °C for 20 minutes.

### PCR

PCR was performed using a Taq polymerase-based fluorogenic assay and primers specific for GAPDH mRNA (Primetime qPCR, Integrated DNA Technologies). Primer sequences used were: 5'-AATCTCCACTTTGCCACTG-3' and 5'CCTCGTCCC GTAGA CAAAA-3'. Reactions were performed using the iCycler (Biorad) as follows: One period of 1.5 minutes at 95°C, followed by 50 cycles of 0.5 minutes at 95°C, 0.5 minutes at 55°C, and 0.5 minutes at 70°C.

### Statistical analysis

The developed Western blot films were photographed using the FluorChem 8900 system (AlphaInnotech). Using the FluorChem 8900 spot densitometry analysis software, a rectangular area was selected which encompassed the region of interest. Selections for each lane were of equal area. The imaging software's automatic background subtraction was used for examining the density of immunostaining for each band. After normalization, statistical analysis was performed as previously described [49, 50]. Welch's modified version of the paired Student's t-test was used to compare means between control and ischemic muscle of each time point, or between the difference in average response between young and old (within each time point). Welch's modified version of the student t-test does not require that the sample variances be equal. Graphs represent the average of individual values (for example, the average GAPDH immunoreactivity of young controls versus the average of young I/R samples, at one day reperfusion). Error bars represent the standard deviation.
